# Demographics and travel history of imported and autochthonous cases of leishmaniosis in dogs in the United States and Canada, 2006 to 2019

**DOI:** 10.1111/jvim.16071

**Published:** 2021-02-26

**Authors:** Taylor Estes Gin, Erin Lashnits, James M. Wilson, Edward B. Breitschwerdt, Barbara Qurollo

**Affiliations:** ^1^ Department of Clinical sciences, College of Veterinary Medicine North Carolina State University Raleigh North Carolina United States; ^2^ Vector‐Borne Disease Diagnostic Lab North Carolina State University Raleigh North Carolina United States; ^3^ Internal Medicine North Carolina State University Raleigh North Carolina United States

**Keywords:** dog, leishmania, lutzomyia

## Abstract

**Background:**

*Leishmania infantum* infections are reported in foxhounds throughout the United States (US) and Canada, but only rarely in other dog breeds. A seroprevalence report from 2006 documented leishmaniosis in foxhounds (8.9%) tested in the US between 2000 and 2003. All other breeds were seronegative.

**Objective:**

To reexamine demographics and travel history of *L. infantum*‐infected dogs in the US and Canada, we hypothesize detection of *L. infantum* in more foxhounds than nonfoxhounds and that infected nonfoxhounds will have traveled to endemic regions.

**Animals:**

A total of 125 dogs positive for *L. infantum* by immunofluorescent antibody, PCR, or both.

**Methods:**

Retrospective, descriptive study of *L. infantum*‐infected dogs between 4 January 2006 and 22 May 2019. Travel history and known lineage to foxhounds was collected from questionnaires.

**Results:**

*Leishmania infantum* was detected in 125 (6.4%) of 1961 dogs tested between 4 January 2006 and 22 May 2019, of which 10 (8%) were foxhounds and 115 (92%) were nonfoxhound breeds. Travel history available for 69 (55%) dogs showed 60 (86.9%) dogs had traveled outside of the US or Canada. Nine (13%) dogs had not traveled outside of the US or Canada, 5 of which were nonfoxhounds.

**Conclusions and Clinical Importance:**

The majority of *L. infantum* cases were detected in nonfoxhounds, many of which had traveled to *L. infantum*‐endemic countries, and several nonfoxhound breeds had no travel history. *Leishmania* surveillance should be considered for dogs that return from *L. infantum*‐endemic regions to monitor emergence of this zoonotic disease in the US and Canada.

**Abbreviations**
CVM: College of Veterinary Medicine
DNA: deoxyribonucleic acid
EDTA: ethylenediaminetetraacetic acid
IFAT: immunofluorescent antibody test
NCSU: North Carolina State University
PBS: phosphate‐buffered saline
rRNA: ribosomal ribonucleic acid
*sod*: superoxide dismutase
VBDDL: Vector‐borne Disease Diagnostic Lab
US: United States

## 1 INTRODUCTION

*Leishmania infantum* causes leishmaniosis in dogs and visceral leishmaniasis in humans throughout the world.^1^ *Leishmania* is transmitted by female phlebotomine sandflies, where endemicity depends on geographic location, climate, reservoir hosts, and competent vectors.^2^, ^3^, ^4^ Leishmaniosis in dogs caused by *L. infantum* has been documented in foxhounds in the United States (US), with the first reported case in Oklahoma in 1980.^5^, ^6^, ^7^, ^8^ A serosurvey of *L. infantum*, primarily in foxhounds sampled between 2000 and 2003 in the US and Canada, reported an overall seroprevalence of 8.9%.^9^ Infection with *L. infantum* has been documented by case reports in nonfoxhounds in the US,^8^, ^10^, ^11^, ^12^, ^13^ but *L. infantum* was not definitively identified in nonfoxhounds in 2 US serosurveys.^9^, ^14^ A recent PCR‐based survey conducted over 9 years on hunting hounds reported an average *L. infantum* prevalence of 20%, but specific breeds were not specified.^15^ Given the lack of known, competent sandfly vectors in the US and evidence supporting vertical transmission in foxhounds, the latter is widely accepted as the only autochthonous method of *L. infantum* transmission within the US, primarily occurring in foxhounds.^16^, ^17^

Dogs could serve as epidemiological reservoirs for *Leishmania* emergence in the US and Canada as climate changes occur and sandfly ranges expand. The South American vector *Lutzomyia anthrophora* was predicted to migrate north through the US by 2020 based on environmental niche modeling.^18^ It is conceivable that sandfly vectors transmitting *L. infantum* (also known as *L. chagasi*) could migrate northward as well.^19^ A study showed that infected US foxhounds can serve as reservoirs for *L. infantum* via *Lu. longipalpis*, a known *L. infantum* vector native to Central and South America.^20^ *Lutzomyia shannoni*, a sandfly native to Georgia, US acquired *L. infantum* after exposure to dogs with leishmaniosis, but it is unknown if *Lu. shannoni* can transmit *L. infantum* to another host.^21^

*Leishmania infantum* screening is not required for dogs entering the US.^22^ Because dogs are routinely imported into the US, consideration should be given to the risk they pose as reservoirs for leishmaniosis.^18^, ^23^, ^24^, ^25^ To our knowledge, no surveys have evaluated the number of *L. infantum*‐infected dogs imported into the US and Canada from *Leishmania*‐endemic countries, only case reports are available.^26^, ^27^, ^28^ Updated demographics on dogs infected with *L. infantum* in the US and Canada, which may not be confined to foxhounds, will inform regulatory agencies and veterinarians of a potentially increasing group of *L. infantum* reservoirs.

We aimed to describe demographic data and travel history for dogs with positive *L. infantum* serology, PCR, or both from a veterinary diagnostic laboratory. We hypothesized that *L. infantum* would be detected in more foxhounds than nonfoxhounds in the US and Canada, and that *L. infantum*‐positive nonfoxhounds would have travel histories to endemic regions.

## 2 METHODS

### 2.1 Dogs

**FIGURE 1:**
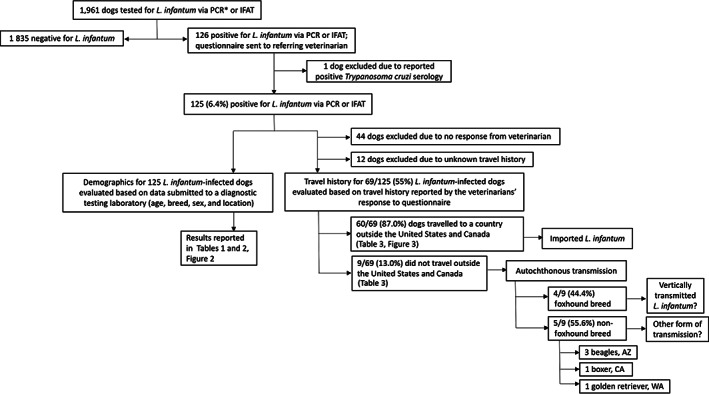
Flowchart outlining inclusion/exclusion criteria for dogs analyzed in this study based on *Leishmania infantum* diagnostic testing results and veterinarians' responses to questionnaire. *All PCR amplicons were sequenced to confirm *L. infantum* infection

Figure 1 provides an overview of the inclusion and exclusion criteria for dogs in our study. After completion of diagnostic testing, sample ownership was transferred to North Carolina State University (NCSU), College of Veterinary Medicine (CVM), Vector‐borne Disease Diagnostic Laboratory (VBDDL) according to terms of the service contract, where we reserved the right to use archived samples for research purposes, with anonymity of the animal, owner, and veterinarian. The laboratory database containing canine vector‐borne disease diagnostic testing results was searched to identify dogs tested for *L. infantum* by PCR, serology, or both between 4 January 2006 and 22 May 2019. All dogs with ≥1 positive test results were included in the study and used to report demographics. Thirteen of the 125 positive dogs were tested more than once, but each dog was only counted as 1 dog in the analysis and considered either PCR negative, positive, or not tested, and IFAT negative, positive, or not tested. Dogs negative for *L. infantum* or dogs that were reported to be positive for *Trypanosoma cruzi* were excluded from analysis because of the potential for cross‐reactivity with *L. infantum* IFAT. For each dog positive for *L. infantum* by PCR or serology, attempts were made to contact the veterinarian on record by email. When email information was not provided, or when the referring veterinarian failed to respond to 2 emails, attempts were made to contact the clinic by phone. Dogs with veterinarians who did not reply to the questionnaire were excluded from analysis of travel history but not the study in general (Figure 1).

### 2.2 Questionnaire and demographic data

Data were obtained and reviewed from the VBDDL database. Data included age, breed, and sex as reported by the referring veterinarian, location within the US or Canada, and date of sample submission for each dog. Region of sample origin was defined based on the US Census Bureau.^29^ Additional data were collected using a questionnaire (Appendix S1) by email or phone contact with the primary veterinarian. Epidemiological information requested included known travel history outside of the US, reason for testing, clinical signs of leishmaniosis, whether the dog was simultaneously tested for *T. cruzi*, and familial ties to a foxhound bloodline. Veterinarians were interviewed about case history by phone or email for nonfoxhounds without travel history to obtain additional details about this unique subset of dogs.

### 2.3 Immunofluorescent antibody test for *Leishmania infantum*

Serial, 2‐fold dilutions of canine sera were made in phosphate‐buffered saline (PBS) containing 0.05% Tween 20, 0.5% nonfat dry milk, and 1% normal goat serum (Fisher Scientific, Gibco cat# PCN5000) before adding 8 to 10 μL to slide wells prepared with cultured *L. infantum* promastigotes, originally isolated from a Foxhound (NCSU‐CVM‐VBDDL *L. infantum*‐Signal strain‐2000‐CO‐1). Slides were incubated in a humidified chamber at 37°C for 30 minutes and washed in PBS at room temperature, at 300 *g*, for 30 minutes. Slides were air‐dried before adding 8 to 10 μL of a 0.01 mg/mL solution of fluorescein isothiocyanate (FITC) goat anti‐dog immunoglobulin G (H&L) conjugate (Sigma cat. # SAB3700115) to each well. Slides were incubated in a humidified chamber at 37°C for 30 minutes before being washed in approximately 400 mL PBS at room temperature, in the dark at 300 *g*, for 20 minutes. Slides were washed for an additional 10 minutes after adding 3 to 4 drops of a 1.65% solution of eriochrome black T counterstain (Sigma cat. 858390). Slides were rinsed with deionized water and dried in the dark before adding a coverslip with antifading mounting medium, Vectashield (Vector Laboratories, ref# H‐100). Slides were evaluated using a fluorescence microscope with exciter and barrier filters (ZEISS Axio Lab.A1, Carl Zeiss Microscopy, Germany) under ×400 magnification. Each slide contained canine seroreactive *L. infantum*‐positive control serum and canine nonreactive negative control serum. In seroreactive samples, parasites were seen as sharply defined promastigotes outlined by distinct green fluorescence. In nonreactive sera, parasite outlines did not fluoresce. Canine sera were screened at 1:16, 1:32, and 1:64 dilutions, and all sera reactive at a titer of 1:64 were repeated and titered to an end‐point titer of 1:8192. To avoid confusion with possible nonspecific binding found at low dilutions, a cutoff titer of ≥1:64 was used to define a seroreactive titer.

### 2.4 PCR for *Leishmania infantum*

Between the years 2006 and 2016, in the VBDDL, *Leishmania* PCR testing was performed using a conventional PCR assay that amplified a 550 bp of the *Leishmania* 18 seconds ribosomal ribonucleic acid (rRNA) gene using a previously described protocol.^5^ During 2016, *Leishmania* 18 seconds rRNA gene PCR testing was replaced by 2 quantitative PCR (qPCR) assays, including a highly sensitive screening assay that amplified a conserved region of minicircle kinetoplast deoxyribonucleic acid (kDNA) and a second assay that amplified the superoxide dismutase (*sod*) gene. Amplicon sequencing of the 18S rRNA gene or the *sod* gene by GENEWIZ, Inc. (Raleigh, North Carolina) was routinely performed at the time of detection. Sequences were compared among other *Leishmania* sp. to verify *L. infantum* infection in our samples. The DNA was extracted from ethylenediaminetetraacetic acid (EDTA) anti‐coagulated whole blood or tissue aspirate samples submitted for *Leishmania* PCR testing. After 2013, to potentially increase PCR sensitivity and decrease cost associated with independent testing of 2 sample types, veterinarians were routinely advised by the VBDDL to add lymph node aspirate material to the EDTA‐whole blood being submitted for *Leishmania* PCR testing. Extractions were performed using a QIAsymphony SP robot (QIAGEN, Valencia, California) and QIAsymphony DNA Mini Kit (QIAGEN, Valencia, California; catalog no. 931236) or Qiagen BioRobot M48 Robotic Workstation with MagAttract DNA Mini M48 kit (Qiagen) depending on the time of sample submission. The absence of PCR inhibitors was demonstrated by amplifying glyceraldehyde‐3‐phosphate dehydrogenase (GAPDH).^30^ Primers for qPCR assays included: LEISH‐KIN‐F3 (5′ CCT CCG GGT AGG GGC GTT C 3′) and LEISH‐KIN‐R (5′ CCT ATT TTA CAC CAA CCC CCA G 3′) or LEISH‐KIN‐R.01 (5′ CCA CCC GGC CCT ATT TTA CAC CAA 3′) for the kDNA assay, and LEISH‐SOD‐F (5′ CCA GAT TCG CGT GCA CTA CG 3′) and LEISH‐SOD‐R (5′ GTT GTT GTA GAC GCC CTT CAG 3′) for the *sod* assay. The PCRs contained 12.5 μL SYBRGreen Supermix (Bio‐Rad, Hercules, California), 5 μL DNA template, primers at 0.3 μM final concentration, and molecular grade water to a final volume of 25 μL. Thermocycler conditions included 98°C for 3 minutes, followed by 40 cycles at 98°C for 15 seconds, 67°C for 15 seconds and 72°C for 15 seconds. Melting temperature (*T*_m_) measurements were made between 65°C and 90°C at 0.5 second intervals where positive *L. infantum* melting temperatures included 84.5°C (kDNA) or 87°C (*sod*). All qPCRs included a positive control consisting of either a previously characterized *L. infantum*‐ or *L. guyanensis*‐infected sample, *Leishmania* kDNA plasmid DNA or *sod* plasmid DNA; and 2 negative controls including a no‐template control consisting of filter‐sterilized, molecular‐grade water and an uninfected canine genomic DNA control.

### 2.5 Retrospective testing to exclude potential *Trypanosoma cruzi*‐infected dogs

Because antibodies against *T. cruzi* can cross‐react with *L. infantum*, retrospective *Leishmania* PCR was performed on archived EDTA whole blood samples from *L. infantum* seropositive dogs that were only tested by IFAT and resided in the West South Central region of the US, where *T. cruzi* is prevalent.^31^, ^32^, ^33^ Archived serum was not available for retrospective *T. cruzi* serology testing.

### 2.6 Statistics

**FIGURE 2:**
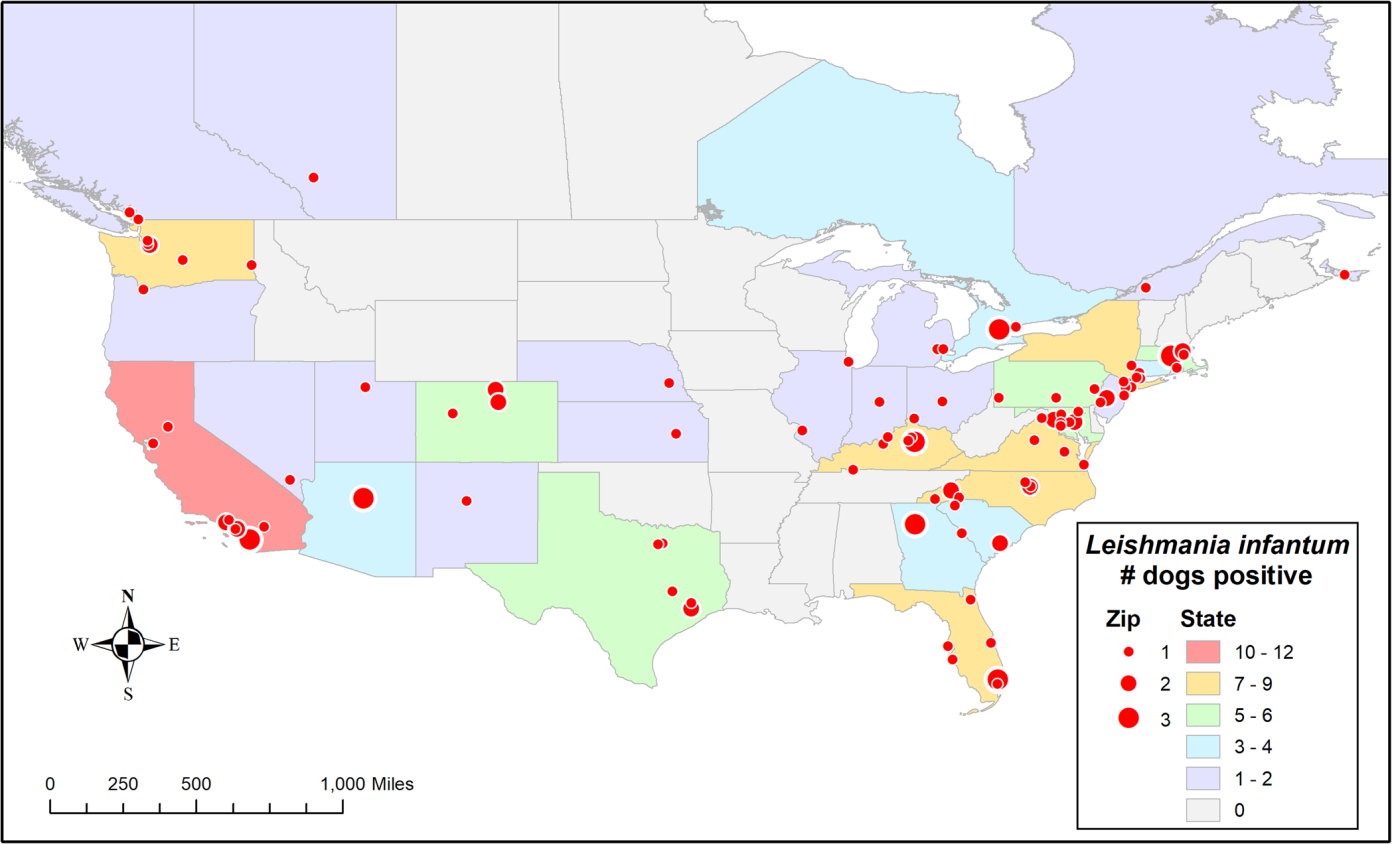
Heat map 1: The colors in this heat map are used to represent locations within the US and Canada where canine samples submitted to one veterinary vector‐borne diagnostic disease laboratory were positive for *Leishmania infantum* either by PCR or IFAT. Numbers next to circles indicate number of positive dogs in a particular zip code

**FIGURE 3:**
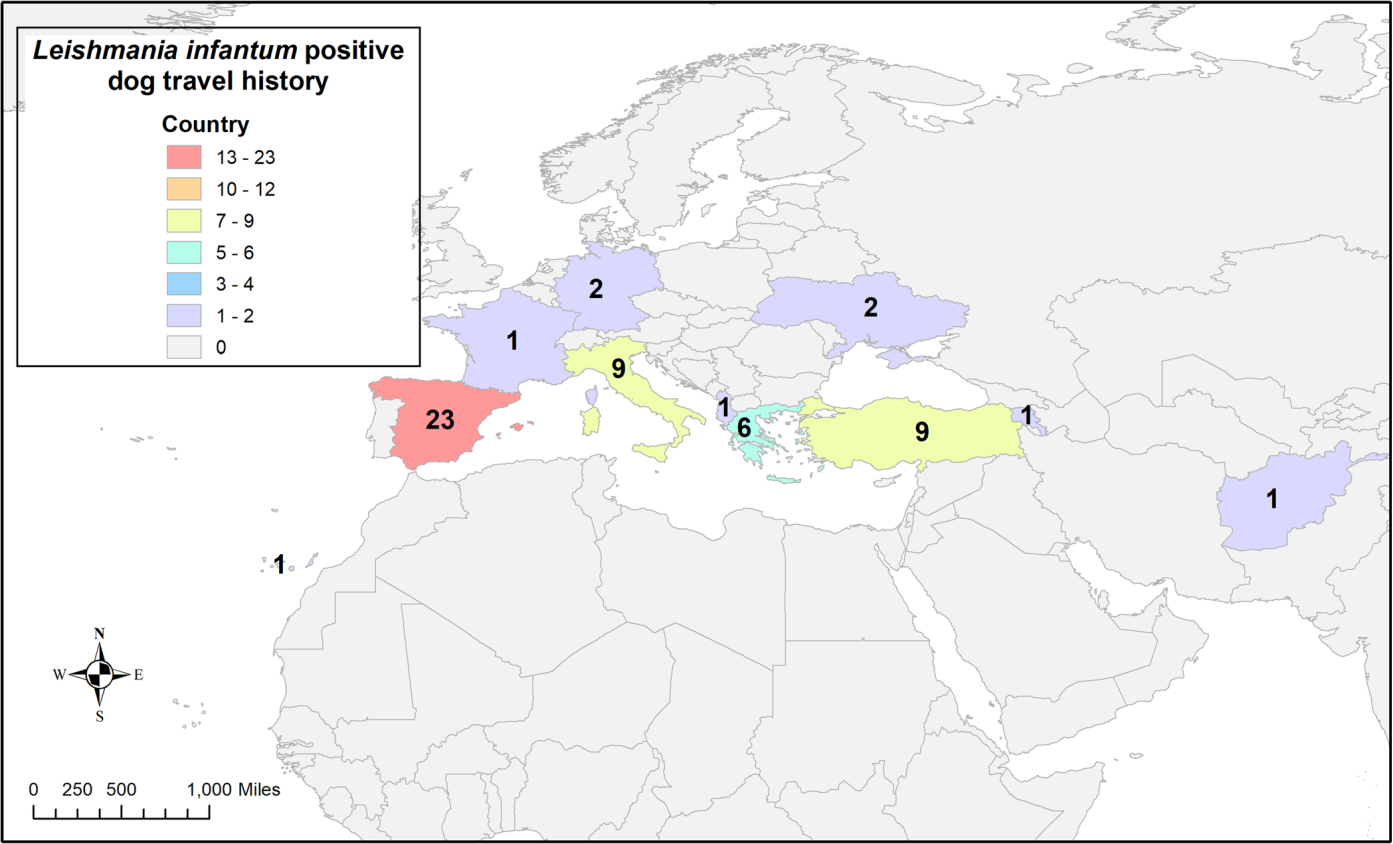
Heat map 2: The colors in this heat map are used to represent locations outside of the US or Canada where dogs with positive *Leishmania infantum* PCR or IFAT as diagnosed at one veterinary vector‐borne disease diagnostic laboratory had reported travel history before the time of *Leishmania* testing. Mexico and Australia are not pictured

Descriptive statistics were used to calculate percentages, means, and ranges, when appropriate. Maps were created using ArcGIS (ArcMap v. 10.5.1, Environmental Systems Research Institute, Redlands, North Carolina). Boundaries were created from publicly available data from the US Census Bureau,^29^ Statistics Canada,^34^ and ArcGIS Enterprise (world map),^35^ using the North American Datum (NAD) 1983 geographic coordinate system with Geodetic Reference System (GRS) 1980 spheroid. All maps were created to show the total number of positive dogs aggregated by state and zip code as reported by the referring veterinarian (Figure 2), and all locations of travel reported by the referring veterinarian were aggregated by country (Figure 3).

## 3 RESULTS

### 3.1 Demographics of study group

During the study period, 1961 dogs within the US (n = 1864) or Canada (n = 97) suspected of having leishmaniosis were evaluated for *L. infantum* by IFAT, PCR, or both in samples submitted for testing by veterinarians. One hundred and forty‐eight breeds were represented with the most prevalent breeds including mixed breed (239/1961, 12.2%), golden retriever (219/1961, 11.2%), German shepherd dog (177/1961, 9.0%), Labrador retriever (116/1961, 5.9%), American foxhound (109/1961, 5.5%), greyhound (104/1961, 5.3%), English foxhound (66/1961, 3.4%), and Belgian malinois (63/1961, 3.2%). Other breeds contained <40 dogs or the breed was not specified in the database (84/1961, 4.3%; Appendix S1). Ages ranged from 1 month to 16 years, with a median age of 4 years. There were 589 (30.0%) castrated male dogs, 566 (28.9%) spayed females, 434 (22.1%) intact males, and 273 (13.9%) intact females. Sex was not reported for 99 (5.0%) dogs. The majority of samples were submitted from the West South Central Region (210/1961, 10.7%), and the fewest samples were submitted from the New England Region (70/1961, 35.7%). For 1 dog, the region of sample origin could not be determined.

### 3.2 Demographics and travel history for *L. infantum*‐positive dogs

After excluding 1 dog because of a subsequently reported positive *T. cruzi* serology, 125 (6.4%) dogs positive for *L. infantum* were evaluated further with respect to demographics and available history (Figure 1). Of the 125 *L. infantum*‐positive dogs, 100 (80%) were IFAT seroreactive (end‐point titers ranging from 1:64 to 1:8192), 61 (48.8%) were *L. infantum* PCR positive, where amplicon sequence analysis confirmed *L. infantum* infection, and 35 (28.0%) were both IFAT and PCR positive. Of the *L. infantum* PCR positive dogs, 60 (98.4%) of 61 samples were blood DNA extractions. Only 1 (1.6%) sample was a lymph node aspirate.

Of the 125 *L. infantum*‐positive dogs in the US and Canada, 117 (93.6%) resided within the US and 8 (6.4%) in Canada at the time of testing (Table 1, Figure 2). Regionally, the majority of *L. infantum‐*positive dogs resided in the South Atlantic and Pacific US (Table 1). No considerable difference was observed between sex, and ages ranged from 9 months to 14 years old, with a median age of 4 years. Overall, breeds were evaluated by groups, with the highest *L. infantum* prevalence calculated in the hound, sporting, and mixed groups (Table 1). Within the 125 *L. infantum*‐positive dogs, 43 different breeds were represented with remaining breeds submitted as mixed, hounds or unknown (Appendix S1). Ten of the 125 (8.0%) dogs were foxhounds (English and American foxhounds) and 115 (92.0%) of 125 were nonfoxhounds. Across all breeds, the most frequently represented included: mixed breed (17/125; 13.6%), greyhound (13/125; 10.4%), golden retriever (10/125; 8.0%), Labrador retriever (8/125; 6.4%), beagle (6/125; 4.8%), English foxhound (6/125; 4.8%), American foxhound (4/125; 3.2%), and unknown (7/125; 5.6%; Table 2). Proportions for other breeds that could be classified as hunting hounds included basset hound (2/125; 1.6%), Ibizan hound (2/125; 1.6%), and dogs specified as hound (2/125; 1.6%) by the submitting veterinarian (Table 2). All other breeds contained ≤4 *L. infantum‐*positives per group.

**TABLE 1:** Demographic data for *Leishmania infantum*‐positive dogs residing in the US or Canada

	IFAT seroreactive	PCR positive	Total *L. infantum* positive
	%, (n)/1516	%, (n)/678	%, (n)/1961
Sex			
F	0.7 (10)	0.9 (6)	0.7 (13)
FS	2.1 (32)	2.8 (19)	2.2 (43)
M	1.2 (18)	1.6 (11)	1.1 (21)
MC	2.3 (35)	3.1 (21)	2.3 (44)
UNK	0.3 (4)	0.3 (2)	0.2 (4)
Breed			
Herding	0.4 (6)	0.9 (6)	0.5 (9)
Hound	2.2 (33)	2.8 (19)	2.1 (41)
Mixed	0.8 (12)	1.5 (10)	0.9 (17)
Nonsporting	0.1 (2)	0.3 (2)	0.2 (3)
Sporting	1.3 (21)	1.6 (11)	1.3 (25)
Terrier	0.3 (4)	0.1 (1)	0.2 (4)
Toy	0.4 (6)	0.4 (3)	0.4 (8)
FSS	0	0	0
Unclassified	0	0	0
Unknown	0.3 (5)	0.6 (4)	0.4 (8)
Working	0.6 (9)	0.4 (3)	0.5 (10)
Submission region			
Canada	0.4 (6)	0.4 (3)	0.4 (8)
East North. Central	0.5 (7)	0.6 (4)	0.4 (7)
West North Central	0.1 (2)	0.1 (1)	0.1 (2)
East South Central	0.3 (5)	0.9 (6)	0.4 (8)
West South Central	0.3 (5)	0.4 (3)	0.3 (5)
Mid‐Atlantic	0.8 (12)	1.3 (9)	0.8 (16)
Mountain	0.7 (11)	0.4 (3)	0.6 (11)
New England	0.4 (6)	0.6 (4)	0.5 (10)
Pacific	1.1 (17)	1.3 (9)	1.0 (20)
South Atlantic	1.8 (28)	2.7 (18)	1.9 (38)
Unknown	0	0	0

*Note*: Sex distribution, breed group, and region of sample origin for dogs that were *L. infantum* indirect fluorescent antibody test (IFAT) seroreactive, PCR positive, or both. % indicates proportion of positive dogs in that category out of all dogs tested by that modality. *US Census Bureau Divisions: New England—CT, ME, MA, NH, RI, VT; Middle Atlantic—NJ, NY, PA; East North Central—IN, IL, MI, OH, WI; West North Central—IA, KS, MN, MO, NE, ND, SD; South Atlantic—DE, DC, FL, GA, MD, NC, SC, VA, WV; East South Central—AL, KY, MS, TN; West South Central—AR, LA, OK, TX; Mountain—AZ, CO, ID, NM, MT, UT, NV, WY; Pacific—AK, CA, HI, OR, WA*.

Abbreviations: F, female; FS, female spayed; FSS, Foundation Stock Service; M, male; MC, male castrated; UNK, unknown.

**TABLE 2:** Breed distribution for *Leishmania infantum*‐positive dogs residing in the US or Canada

	Tested^a^ for *L. infantum*	*L. infantum* positive^b^
	%, (n)/1961	%, (n)/125
Basset hound	1.5 (29)	1.6 (2)
Beagle	1 (20)	4.8 (6)
Foxhound^c^	8.9 (175)	8 (10)
Golden retriever	11.2 (219)	8 (10)
Greyhound	5.3 (104)	10.4 (13)
Hound	1.4 (28)	1.6 (2)
Labrador retriever	5.9 (116)	6.4 (8)
Mixed	12.2 (239)	13.6 (17)
Unknown	4.3 (84)	5.6 (7)

*Note*: % indicates proportion of dogs tested in that category out of all dogs tested (n = 1961), or proportion of positive dogs in that category out of all positive dogs (n = 125).

^a^ Dogs were tested by *L. infantum* indirect fluorescent antibody test (IFAT) or PCR.

^b^ Dogs were positive by *L. infantum* IFAT or PCR or both.

^c^ Foxhounds include American foxhounds and English foxhounds.

Contact was attempted with the recorded veterinary clinic for all of the 125 *L. infantum*‐positive dogs evaluated, and responses were received for 81 (64.8%) dogs. For 12 of these 81 dogs, answers regarding travel history outside of the US or Canada were not available from the clinical record for various reasons. Travel histories for the remaining 69 dogs are summarized in Table 3. Of the 69 dogs with available travel history, 9 (13.2%) dogs had never traveled outside of the US or Canada. Four foxhounds and 5 nonfoxhounds had no travel history to endemic areas (Figure 1). Of the nonfoxhounds without travel history, 3 beagles were from Arizona, 1 boxer was from California, and 1 golden retriever was from Washington. Sixty (87%) dogs were reported to have originated from or traveled to countries outside of the US or Canada, most of which are endemic for *L. infantum* (Table 3, Figure 3).

**TABLE 3:** Country of reported travel histories for *Leishmania infantum*‐positive dogs residing in the US or Canada

	IFAT seroreactive	PCR positive	Total *L. infantum* positive
	%, (n)/48	%, (n)/30	%, (n)/69
Spain	43.8 (21)	33.3 (10)	33.3 (23)
Italy^a^	14.6 (7)	13.3 (4)	13.0 (9)
Turkey^a^	16.7 (8)	16.7 (5)	13.0 (9)
Greece	8.3 (4)	6.7 (2)	8.7 (6)
Mexico	4.2 (2)	3.3 (1)	2.9 (2)
Brazil	4.2 (2)	3.3 (1)	2.9 (2)
Germany^a^	4.2 (2)	0	2.9 (2)
Russia^a^	0	6.7 (2)	2.9 (2)
Ukraine^a^	0	6.7 (2)	2.9 (2)
Afghanistan	2.1 (1)	3.3 (1)	1.4 (1)
Armenia	2.1 (1)	3.3 (1)	1.4 (1)
France^a^	2.1 (1)	3.3 (1)	1.4 (1)
Albania	2.1 (1)	3.3 (1)	1.4 (1)
Australia	2.1 (1)	0	1.4 (1)
Turks and Caicos^a^	2.1 (1)	3.3 (1)	1.4 (1)
Canary Islands^a^	2.1 (1)	3.3 (1)	1.4 (1)
Jordan	0	3.3 (1)	1.4 (1)
No travel	16.7 (8)	10.0 (3)	13.0 (9)

*Note*: Country from reported travel histories for dogs from the US or Canada that were *L. infantum* indirect fluorescent antibody test (IFAT) seroreactive or PCR positive. % indicates proportion of positive dogs in that category out of all dogs tested by that modality where an answer was provided for “travel history.”

^a^ Dogs with travel to multiple areas were included for each region of travel.

Protozoal cross‐reactivity on serology, specifically with *T. cruzi*, was considered for dogs in the West South Central region where *T. cruzi* is known to be most common in the US.^31^, ^32^, ^33^ Two dogs from this region were *L. infantum* IFAT positive; stored whole blood was available for PCR on 1 of these 2 dogs. This dog was positive for *L. infantum* by kDNA and *sod* PCR analysis, with amplicon sequence confirmation. Archived serum from these 2 dogs was not available for *T. cruzi* serology testing to evaluate for potential cross‐reactivity.

More in‐depth clinical information was obtained by phone or email with the primary veterinarian for *L. infantum*‐positive nonfoxhounds without travel history. The goal of further investigating these dogs was to ascertain details about the route of infection, because autochthonous transmission of *L. infantum* is rare in nonfoxhounds in the US. The 3 beagles were reported to live in a hunting kennel. Several older dogs were housed in the kennel, including the beagles we tested, with unexplained kidney failure and weight loss. Necropsy with histopathology failed to identify an underlying cause. Ultimately, these 3 beagles and many other dogs in the kennel were tested for *L. infantum*. All 3 beagles were IFAT positive (1:512, 1:1024, and 1:1024). Only 1 beagle, with IFAT 1:1024, was tested by PCR and found to be negative. Although not the beagles in our report, several other dogs in the same kennel underwent some form of *T. cruzi* testing and were negative (tests were ordered by the primary veterinarian and results reported as negative). The primary veterinarian reported no travel history to *Leishmania*‐endemic regions for any of the beagles in the kennel, and no foxhound bloodlines were interwoven within the beagle lines. The kennel tested all of their other hounds for *L. infantum*, which included foxhounds, sighthounds, and hound mixes, and all were negative. Coccidiomycosis was found in some dogs in the pack, which the primary veterinarian reported as common in the area. The boxer in Sacramento, California, was IFAT positive at 1:2048; PCR testing was not performed. This information recently was published as a case report.^13^ The referring veterinarian reported that this dog was tested because of the presence of protozoal organisms consistent with *Leishmania* on cytology of an enlarged lymph node (location of node not specified). The dog originally presented for lymphadenopathy, as well as a diagnosis of systemic histiocytosis based on histopathology of a skin biopsy (site not specified) performed to evaluate crusting cutaneous lesions. The dog had no travel history outside of the US, but the owners were reported to be from Europe (Spain and Germany). The dog was not tested for *T. cruzi*. The golden retriever presented to a specialty referral hospital for evaluation of anemia and chronic weight loss. The dog historically had been diagnosed with suspected immune‐mediated hemolytic anemia and placed on immunosuppressive drugs several months earlier. The dog also had a history of intermittent epistaxis, positive nasal fungal culture (commensal yeast, species not indicated), positive *Babesia canis* titers, and bilateral cruciate ligament ruptures. On presentation at the time of *L. infantum* diagnosis, the dog was anorexic, had muscle wasting, and was found to be moderately hyperglobulinemic and mildly anemic with positive slide agglutination testing and spherocytes. After failing to respond to medical treatment, the dog was taken to surgery for liver biopsy, splenectomy, and lymph node biopsy. The dog had histiocytic inflammation with protozoal organisms in the spleen, most consistent with *L. infantum*. No organisms were found in the canine liver or lymph nodes. Blood was sent to the VBDDL for *L. infantum* testing, and the dog was *L. infantum* PCR positive and IFAT positive at 1:1024. *Trypanosoma cruzi* testing was not performed. The dog reportedly was moved to Washington from Kentucky at the age of 2 months and had otherwise never traveled outside of Washington.

## 4 DISCUSSION

Of the 1961 dogs residing in the US or Canada that were tested for *L. infantum*, 125 (6.4%) were positive (either by IFAT, PCR or both assays), with 92% being breeds other than foxhounds. At least 48% of all positive dogs (82.6% of positive dogs with answers reported for “travel history”) were imported from endemic countries. Nine of the *L. infantum*‐positive dogs in our study had no known travel history outside of the US or Canada, of which 4 were foxhounds and, surprisingly, 5 were nonfoxhounds. These included 5 dogs with either clinical syndromes or cytology or biopsy results compatible with *L. infantum*: 3 IFAT positive beagles in a hunting kennel in Arizona, 1 IFAT positive boxer in California, and 1 PCR and IFAT positive golden retriever in Washington.

In the US and Canada, few reports have described *L. infantum* in nonfoxhound breeds lacking travel history since 1991, including a basenji in Texas (1991), a toy poodle in Maryland (2000), a Newfoundland in Pennsylvania (2000), a spinone Italiano in North Carolina (2001), a beagle and its housemate (breed not reported) in Alabama (2001), a Doberman pinscher in Massachusetts (2001), and most recently a boxer in California (2020).^8^, ^10^, ^11^, ^12^, ^13^ In most of these cases, except for the basenji and the toy poodle, which were seemingly autochthonous, exposure to *L. infantum* was traced back to other *L. infantum*‐positive dogs, but definitive direct transmission was never documented. In the case of the boxer, there was no known travel history for the positive dog itself, but the bitch to which it was borne was imported from Spain, thus vertical autochthonous transmission was likely.^13^

Vertical autochthonous transmission in dogs is well documented and is thought to be the primary mechanism by which foxhounds in the US transmit *L. infantum*.^16^, ^17^, ^36^, ^37^ This mechanism likely explains many of the positive tests observed in the foxhounds in this sample, especially in foxhounds with no travel history outside of the US. Although vertical autochthonous transmission is theoretically possible in nonfoxhounds without travel history, particularly in breeds kenneled with foxhounds where transmammary transmission may occur, additional mechanisms for transmission must be considered in nonfoxhounds, such as vector‐borne or direct transmission (inoculation of wounds or by blood transfusion). To our knowledge, only 2 serosurveys have been conducted (2003, 2006) to assess the prevalence of *L. infantum* antibodies in nonfoxhounds in the US, and neither reported *L. infantum* in nonfoxhounds, even in dogs that resided in the same kennel as the foxhounds.^9^, ^14^ Recently, a study assessed *L. infantum* in hunting hounds in the US, reporting a high prevalence of infection (≥20%), but it was not clear if breeds other than foxhounds were infected.^15^ Additional prospective studies are needed to further define if breeds other than foxhounds in the US and Canada are infected through vertical, direct, or vector‐borne autochthonous transmission.

Although transmission competence of *L. infantum* by an indigenous US sandfly species has not been documented, *Lu. shannoni*, a sandfly identified in the southeastern US, can become infected with *L. infantum* when experimentally fed on dogs infected through vertical transmission, demonstrating theoretical potential to serve as a competent vector in the US.^21^, ^38^ Furthermore, dogs have the potential to serve as epidemiological reservoirs for *L. infantum* emergence in the US as climate changes occur and sandfly ranges expand northward.^38^ A 2011 study using multilocus microsatellite typing showed that the South and Central American strains of *L. infantum* originated from dogs imported from Europe and the Mediterranean.^39^ Indigenous sandflies such as *Lu. longipalpis* s.l. adapted to transmit *L. infantum* and this species is now the major sandfly vector of *L. infantum* in the Americas.^40^ These findings emphasize the diversity of *Lutzomyia* spp. vectoral capacity for *L. infantum* and support the hypothesis that *Leishmania* spp. and *Lutzomyia* spp. coevolve, changing *Leishmania* transmission dynamics.^41^, ^42^, ^43^ Further investigation and ongoing surveillance are warranted to determine if indigenous North American sandfly species such as *Lu. shannoni* could coevolve to transmit *L. infantum* in the US and Canada, or if vector competent species from Central America expand northward into the US as climate changes occur.

Our study indicates that at least 48% of all of the *L. infantum*‐infected dogs in the US positive for *L. infantum* were imported from endemic areas. One important group is imported rescued dogs, including golden retrievers from Turkey and greyhounds originating from Spain, a country where this breed is routinely used in racing and hare‐hunting competitions.^44^ Although rescue is an important humanitarian effort, benefits of importing dogs infected with *L. infantum* should be balanced against the risk of developing endemic foci of transmission in the US and Canada. In the context of One Health (animals, humans and the environment), dogs from *L. infantum*‐endemic regions should be screened before or upon US entry, as is done in other countries, and a national surveillance database of *L. infantum‐*positive dogs created as a public health consideration. Despite the very close proximity of dogs to humans, exposure to many shared vectors, and the acquisition of the same vector‐borne diseases, there is no federal monetary support for disease surveillance among companion animals, as occurs for production animals (US Department of Agriculture) and humans (Centers for Disease Control and Prevention).^25^ Combined, these facts highlight the importance of screening dogs imported into the US either by rescue groups, the military, or companion animal travel. *Leishmania infantum*‐exposed or infected dogs can and should then be treated and provided appropriate sandfly repellent products to limit the risk *L. infantum* transmission poses to humans and other dogs.

Our study had several limitations. Ours was a retrospective analysis utilizing a set of convenience samples and thus does not represent a seroprevalence study. One potential source of bias includes the subset of positive nonfoxhounds without travel history, wherein veterinarians were contacted for additional information about the case. Doing so generated a more detailed understanding of these cases as compared with cases where the veterinarian provided limited information in the questionnaire. Additionally, given that demographics were reported by the primary veterinarian, the results may not entirely reflect the true breed distributions if breeds were reported inaccurately. Referring veterinarians also were called or emailed to answer the questionnaire, with some of the visits dating back several years. This creates the opportunity for recall bias, especially in the context of reporting clinical signs, travel history, original reason for sample submission, or some combination of these. Furthermore, information about potential blood transfusions or dog bites was not obtained for all of the *L. infantum*‐positive dogs. In the context of PCR sample sensitivity, submitted blood samples may or may not have had lymph node aspirate material added, beginning after 2013, creating variable sensitivity.^45^ Overall, *Leishmania* PCR has been reported to be 100% specific,^45^ indicating that a positive PCR with amplicon sequencing in this population likely reflects a true *L. infantum*‐positive. Specificity of *L. infantum* IFAT, however, is lower (approximately 91%‐99%) and has the potential for cross‐reactivity increasing the probability for false positives specifically in the population of IFAT positive and PCR negative (or PCR untested) dogs.^46^

Antibodies against *T. cruzi* potentially can cross‐react with *Leishmania*.^47^ Only 1 dog that we were aware of underwent testing to identify *T. cruzi* exposure: a hound from the West South Central region (Texas). The dog was reported to be PCR negative for *Leishmania* by the primary veterinarian, with positive *T. cruzi* and *L. infantum* serology. This dog ultimately was excluded from analysis because of concern for cross‐reactivity. The West South Central region (Texas, Louisiana, Oklahoma and Arkansas) was a focus of possible *T. cruzi* cross‐reactivity because of the greater distribution of Chagas disease in the Southern US where its associated vector and reservoirs are found.^48^ The remaining *L. infantum*‐positive dogs from the West South Central region included 4 PCR and IFAT positive dogs, as well an IFAT positive dog where PCR testing was not performed. Although it is possible, this dog was only exposed to *T. cruzi*, additional evidence supported an infection with *L. infantum*, including clinical signs consistent with leishmaniosis and a history of travel to a *L. infantum* endemic country (Spain) before testing. Considering that over half of the *L. infantum*‐positive dogs in our study were confirmed by PCR and sequence analyzes, and the remaining dogs tested did not reside in or travel to regions with a high prevalence of *T. cruzi*, we believe it is unlikely that cross‐reactivity substantially influenced the outcome of our study.

In conclusion, we report that *L. infantum* was detected in more nonfoxhounds than foxhounds residing in the US and Canada, and that many of these dogs had traveled to, or been imported from, endemic regions. Our study also emphasizes the need for clinical veterinarians to obtain good travel histories, screen for *L. infantum* in dogs with travel histories to endemic regions and consider *L. infantum* in breeds other than foxhounds. We also identified 5 nonfoxhounds that had no travel history outside of the US and no known source of vertical transmission. This finding emphasizes the importance of *L. infantum* surveillance within the US and Canada so as to monitor the potential for endemic transmission. If vector‐borne transmission is occurring on rare occasions in dogs in the US and Canada, it undoubtedly would have important public health implications for humans.

## OFF‐LABEL ANTIMICROBIAL DECLARATION

Authors declare no off‐label use of antimicrobials.

## CONFLICT OF INTEREST DECLARATION

B.A. Qurollo, codirector of the NC State‐VBDDL and IDEXX Laboratories, funds a portion of her salary. E.B. Breitschwerdt codirects the NC State‐VBDDL and the Intracellular Pathogens Research Laboratory at NC State, is chief scientific officer at Galaxy Diagnostics and a paid consultant for IDEXX Laboratories, Inc. T.E. Gin, E.L. Lashnits, and J.M. Wilson have no competing interests to declare.

## INSTITUTIONAL ANIMAL CARE AND USE COMMITTEE (IACUC) OR OTHER APPROVAL DECLARATION

Authors declare no IACUC or other approval was needed as this is a retrospective article.

## HUMAN ETHICS APPROVAL DECLARATION

Authors declare human ethics approval was not needed for this study.

## DATA AVAILABILITY STATEMENT

The data that support the findings of this study are openly available in Mendeley Data at https://doi.org/10.17632/cvv3pydj8g.1.

## Supporting information


**Appendix** **S1.** Veterinarian questionnaire.
