# Supplementary material for: Demographics and travel history of imported and autochthonous cases of leishmaniosis in dogs in the United States and Canada, 2006 to 2019
Source: J Vet Intern Med. 2021 Feb 26;35(2):954–64. doi: 10.1111/jvim.16071 (PMC7995368; doi:10.1111/jvim.16071)
Supplement: Supplementary file 1 — Appendix S1. Veterinarian questionnaire. [file JVIM-35-954-s001.pdf]

1) What was the indication for Leishmaniosis testing (routine screening, travel history, history of Leishmoniasis, clinical signs consistent with Leishmoniosis, other)?

2) If testing was due to clinical signs, please briefly describe the patient's signs.

3) Does this patient have any history of travel outside of the US and if so, where?

4) Does this patient have known familial history of the Foxhound bloodline?

5) Was this patient tested for *T. cruzi*?
